# Enhancing the effectiveness of flipped classroom in health science education: a state-of-the-art review

**DOI:** 10.1186/s12909-021-03052-5

**Published:** 2022-01-12

**Authors:** Janique Oudbier, Gerard Spaai, Karline Timmermans, Tobias Boerboom

**Affiliations:** Amsterdam UMC, AMC-University of Amsterdam, Amsterdam, the Netherlands

**Keywords:** Flipped classroom, Effectiveness, Advantages, Disadvantages, Mediators, Interventions, Health science education

## Abstract

**Background:**

In a flipped classroom, students acquire knowledge before class and deepen and apply this knowledge during class. This way, lower-order learning goals are achieved before class and higher-order skills are reached during class. This study aims to provide an overview of the factors that contribute to the effectiveness of the flipped classroom and how these factors can be stimulated. The effectiveness of the flipped classroom is conceptualized in this study as test scores, the achievement of higher learning goals, and student perceptions.

**Methods:**

A state-of-the-art review was conducted. The databases MEDLINE, PsycINFO, PubMed, Web of Science, and Scopus were consulted. The timeframe is 2016 till 2020. The studies were qualitatively analyzed according to the grounded theory method.

**Results:**

After screening the studies based on the inclusion-and exclusion criteria, 88 studies were included in this review. The qualitative analysis of these studies revealed six main factors that affect the effectiveness of the flipped classroom: student characteristics, teacher characteristics, implementation, task characteristics, out-of-class activities, and in-class activities. Mediating factors are, amongst other factors, the learner’s level of self-regulated learning, teacher’s role and motivation, assessment approach, and guidance during self-study by means of prompts or feedback. These factors can be positively stimulated by structuring the learning process and focusing the teacher training on competencies and learning-and teaching approaches that are essential for the flipped classroom.

**Conclusion:**

This paper provides insight into the factors that contribute to the effectiveness of the flipped classroom and how these factors could be stimulated. In order to stimulate the effectiveness of the flipped classroom, the positively and negatively affecting factors and mediating factors should be taken into account in the design of the flipped classroom. The interventions mentioned in this paper could also be used to enhance the effectiveness.

**Supplementary Information:**

The online version contains supplementary material available at 10.1186/s12909-021-03052-5.

## Background

In a flipped classroom, the learning process of the traditional classroom is reversed. During out-of-class activities students acquire knowledge and this knowledge is deepened and applied during in-class activities. In this way, the lower-order learning goals are achieved before class and the higher-order learning goals are reached during class under the guidance of a teacher. Lower order learning goals are for example, the recall of facts and the understanding of information, whereas higher learning goals are amongst other skills the solving of problems and making choices based on the analysis of information [76]. Flipped classroom often uses a blended learning approach. Blended learning integrates different forms of learning, such as face-to-face learning and online learning [28].

In health science education lectures are still a common form of instruction. However, Mehta et al. [58] stated that most current health science education is inefficient, inflexible, and lacks student-centeredness. Furthermore, they underlined that learning is often focused on performance, instead of the development of competencies. Therefore, they mentioned that it is time for a new paradigm for medical education [58]. The need to focus more on educational approaches that support the learner to process information is also underlined by van der Vleuten and Driessen [89]. They consider flipped classroom as a great educational approach to enable student-centered learning [89].

The effectiveness of the educational approach flipped classroom in health science education has been extensively studied in recent years. Hew and Lo [37] have conducted a meta-analysis on the improvement of student learning in health science education using a flipped classroom approach compared to a traditional classroom. They define traditional classroom as follow: “the approach of having students come to class during which teachers use a range of pedagogical strategies (e.g., lecture, case discussion, student presentation), and then students complete most of their homework after school”. Student learning is defined in this study as the difference in performance before and after the flipped classroom ([37], p. 2). This study showed that flipped classroom has a significant positive effect on student learning compared to a traditional classroom, and that students prefer flipped classroom over traditional classroom. Chen et al. [17] have conducted a meta-analysis on the academic outcomes of flipped classroom learning. This study showed that student achievement is higher in a flipped classroom than in a lecture-based classroom. Student achievement in this study is defined as course grades or examination scores.

Although the effectiveness of the educational approach flipped classroom in health science education is studied, a qualitative review that provides an overview of the factors that contribute to its effectiveness and how these factors could be stimulated is lacking. Therefore, this review presents an overview of the factors that contribute to the effectiveness of the flipped classroom, the mediating factors, and interventions to stimulate the effectiveness. These insights can be used to effectively implement the flipped classroom in a curriculum.

### Research objectives

 The aim of this study is to investigate the current state of knowledge about factors contributing to the effectiveness of the flipped classroom and interventions to positively stimulate these factors. The effectiveness of the flipped classroom is conceptualized in this study as test scores, the achievement of higher learning goals, and student perceptions (e.g. motivation, satisfaction, engagement). The main question that will be focused on is: Which factors affect the effectiveness of the flipped classroom and how could these factors be positively stimulated? To answer this question the following sub questions will be answered: 1) Which factors affect the effectiveness of the flipped classroom positively or negatively? 2) Which factors intermediate the effectiveness of the flipped classroom? 3) How could the affecting and intermediating factors be stimulated in a positive way?

Intermediating factors are defined in this study as factors that mediate the effect of the positive and negative factors on the effectiveness of the flipped classroom. An example of an intermediating factor is the inclusion of seductive details. The effect of level of prior knowledge on the effectiveness of the flipped classroom is mediated by the inclusion of seductive details, because the flipped classroom is more effective for students with a high level of prior knowledge when seductive details are included. Affecting factors are conceptualized in this study as positive and/or negative factors that affect the effectiveness of the flipped classroom in a positive and/or a negative way.

## Methods

### Search strategies

 This study is a state-of-the-art-review which is defined by [33] as follow: “ A state-of-the-art review tends to address more current matters in contrast to other combined retrospective and current approaches. This kind of review may offer new perspectives on issue or point out area for future research” ([33], p. 94). At the beginning of April 2020, the databases MEDLINE, PsycINFO, PubMed, Web of Science, and Scopus were consulted. These databases are well-known in the educational and biomedical sciences and comprise a broad selection of articles. The aim was to retrieve all the studies that are conducted on the instructional method flipped classroom in the health science education domain and contribute to answering the research questions, except the studies that are conducted on team-based learning. This decision was made, because team-based learning is a specific and unique form of flipped classroom. Team-based learning relies more heavily on small group teaching than other instructional methods [60]. Team-based learning is furthermore characterized by a sequence of steps: 1) advance assignment, 2) individual readiness assurance test, 3) team readiness assurance test, 4) instructor clarification review, 5) team application, 6) appeal. Another crucial element of team-based learning is peer evaluation [67]. Especially the fact that summative assessment is a part of a team-based learning sequence, makes this educational strategy different from other flipped classroom designs. Because of the uniqueness and specific features, team-based learning is hard to compare with other forms of flipped classroom. Team-based learning has its own line of research that is especially focused on this form of flipped classroom [70].

By consulting the databases, the following search terms were used in titles and abstracts: (flipped classroom) AND (higher education) NOT (team-based learning), (inverted classroom) AND (higher education) NOT (team-based learning), and (flipped learning) AND (higher education) NOT (team-based learning). The time was limited by the last five years (2016 till 2020), because studies within this time frame provide the most current insights.

### Inclusion and exclusion criteria

 Table 1 shows the inclusion and exclusion criteria that are used in this study to include or exclude the articles.

**Table 1:** Inclusion and exclusion criteria

Criteria	Inclusion	Exclusion
Language	The study is published in English	The study is published in a Non-English language
Didactic principle	The main didactic principle is flipped classroom	The main didactic principle is different from flipped classroom, (such as team-based learning)
Participants	Students in higher education	Students in primary or secondary education or adult education
Type of publication	All types of publications, except contributions to conferences, validation studies, and pilot studies.	Contributions to conferences, validation studies and pilots are excluded.
Availability	The full-text of the study must be available to consult via library systems or internet	Studies of which the full-text was not available to consult
Type of education	The type of education is lecture and/or tutorial.	The type of education is clerkship
Answers research question	The article’s purpose and/or research question contributes to answering the main question.	The article’s purpose and/or research question does not contribute to answering the main question.
Research design	The design of the study is mixed method, qualitative or quantitative	The design of the study is descriptive
Domain	Health science education	Other domains than health science education

**Fig. 1:**
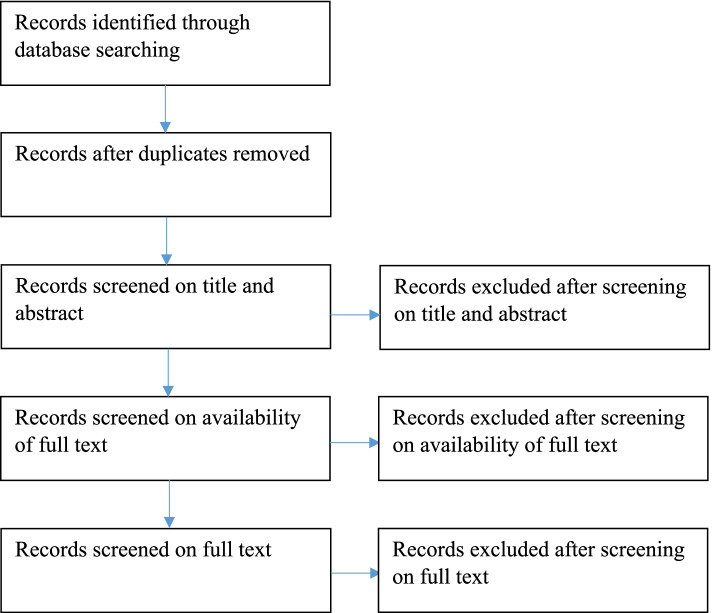
Inclusion-and exclusion procedure

Figure 1 shows the inclusion-and exclusion procedure

Of all records 69.8% (*k* = 1276) was screened by a second researcher (MJO and GS). The percentage of agreement on inclusion/exclusion of studies based on titles and abstracts was 95.49. This was considered satisfactory. Differences were discussed upon consensus was reached.

### Data appraisal

 The methodological quality of the studies was assessed by the checklist for quasi-experimental studies and the checklist for qualitative studies by the Joanna Briggs Institute (JBI, 2020). The quality of 10 randomly chosen articles was assessed by two researchers independently (JO and KT). The total percentage of agreement was 71.38%. The differences were discussed upon consensus was reached.

### Data extraction

 To assess the main features of the studies, the included studies were systematically analyzed by making use of a coding scheme. The coding scheme is displayed in Additional file 1. Using this coding scheme, amongst other features of the studies sample characteristics, educational context features, methodological features, and general study information were assessed for each study. Two researchers independently coded a set of 10 randomly chosen articles (JO and KT). The percentage of agreement was 78.85 on features of the study and differences were discussed upon consensus was reached. This percentage was considered satisfactory.

### Data analysis

 Since the outcome variables, research designs, and research methods differ a lot between the studies, we have decided to narratively summarize the data, rather than conducting a quantitative meta-analysis. The qualitative analysis of the data was conducted according to the grounded theory method described by Boeije (2010). We choose this method, because it enables a more detailed analysis and is applicable for the construction of a new theory. The analysis consisted of two steps: segmenting and reassembling. Segmenting consisted of open and axial coding. Open coding is the fragmentation of text and the labeling of the fragments with codes and axial coding is the establishments of relations between codes, the clustering of codes, and the defining of codes. In the last step of the analysis, reassembling, the core category is determined to which all the other categories can be related. The software package MaxQDA 2020 (VERBI Software, 2019) was used for coding the studies. During the analysis memos were written to write up ideas that arise during coding. The emerging themes were discussed in the research group.

## Results

### Search results

**Fig. 2:**
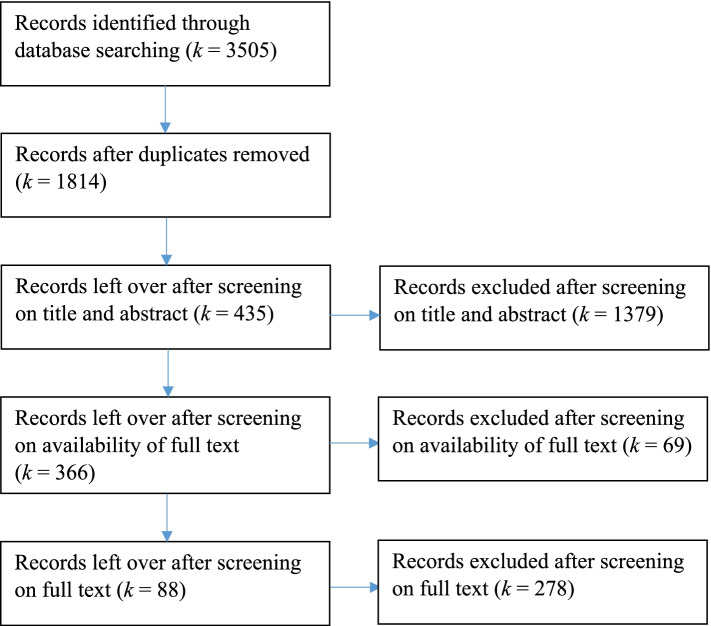
PRISMA Flow Diagram of Literature Search and Processing of Records. Preferred Reporting Items for Systematic Reviews and Meta-analyses

 In total 3505 studies were retrieved. Of these retrieved studies duplicates were deleted by the first author (*k* = 1691). During the first screening of titles and abstracts, studies in which flipped classroom was not the main didactic principle (*k* = 628), for example studies on MOOCs, were excluded from this review. Studies in which higher education was not the population (*k* = 136), for example studies conducted in primary education, were also excluded. Furthermore, contributions to conferences, validation studies and dissertations (*k* = 333) and Non-English articles (*k* = 24) were not included in this study. After the first screening, the accessibility of the full-text of the articles was checked and results were excluded if the full-text could not be obtained (*k* = 69). During the second screening, the full text of the remaining 366 records were assessed for eligibility based on the inclusion and exclusion criteria mentioned in Table 1. After the second screening, 278 results were excluded because they did not meet all of the inclusion criteria from Table 1. Figure 2 shows the records of the literature search and the selection procedure of the articles.

### Study characteristics

 Additional file 1 shows the characteristics of the included studies such as research design, research method, research quality, and topic per study.

### Study quality assessment

 The quality of the studies was overall sufficient, the quality of 11 of the 88 studies was insufficient. The studies with insufficient quality were also included in this study. Additional file 1 shows the study quality assessment.

### Effectiveness of the flipped classroom

 The effectiveness of the flipped classroom is in the included studies often expressed as test scores. Several studies have shown an increase in students’ short-term scores on a summative test in a flipped classroom compared to a traditional classroom [1, 4, 14, 18, 19, 30, 31, 35, 47, 50, 61–64, 73, 78, 85, 87, 95, 99]. However, Chien and Hsieh [19] and Chutinan et al. [21] showed that the long-term effects of the flipped classroom were insignificant, when students returned to a traditional classroom setting. This means that the flipped classroom does not have a long-term effect. Mennella [59] concluded that the flipped classroom does not have benefits regarding test scores over other active-learning components.

Other ways to express the effectiveness of the flipped classroom is student perceptions, amongst other perceptions satisfaction, motivation, and engagement. Several studies have shown an increase in student perceptions [18, 55, 61].

The qualitative analysis of the included studies according to the grounded theory method revealed six main factors that play a role in the effectiveness of the flipped classroom; 1) student characteristics, 2) implementation, 3) task characteristics, 4) out-of-class activities, 5) in-class activities, 6) teacher characteristics. The factor student characteristics consists of the sub-factors 1a) self-regulated learning skills, 1b) prior knowledge, and 1c) learning attitude. Self-regulated learning is the planning, monitoring, and evaluation of learning [100]. Teacher characteristics is conceptualized in this study as the skills, knowledge, attitude, and traits of a teacher.

#### Positive and negative factors contributing to the effectiveness of the flipped classroom

 The first research question addressed the factors which contribute positively or negatively to the effectiveness of the flipped classroom. The analysis showed several factors that affect the flipped classroom negatively or positively. Table 2 shows the factors that affect the effectiveness of the flipped classroom positively or negatively, organized by the aforementioned main factors. The main factor teacher characteristics affects the effectiveness of the flipped classroom in an indirect way as a mediator. How teacher characteristics affect the effectiveness of the flipped classroom will therefore be further described in the next section.

**Table 2:** Positive and negative factors of the flipped classroom

Factors	Positive factors	Negative factors	References
**Student characteristics**			
*Self-regulated learning skills* (1a)	-Prevents procrastination -Learner can learn at his own pace -Learner is responsible for his own learning -Development of self-directed learning readiness -Timely feedback -Learner reflects on his learning	-More self-regulation is required -May lead to non-attendance or non-preparation	[8, 9, 10, 14, 18, 19, 22, 68, 71, 83, 92]
*Prior knowledge* (1b)		-More prior knowledge required	[34]
*Learning attitude* (1c)	Enhancement of -concentration -engagement -motivation -confidence -satisfaction -self-efficacy	-Not all students prefer the flipped classroom -More motivation required	[1, 8, 16, 18, 19, 22, 25, 27, 30, 31, 32, 34, 35, 50, 61, 63, 64, 65, 73, 77, 82, 84, 87, 91]
Implementation (2)		-Time and resources should be invested -In technology should be invested	[1, 15, 19, 68, 71, 83]
Task characteristics (3)	-Learning of higher cognitive skills -Enhancement of problem-solving ability, critical thinking, and decision-making performance -Competence-oriented	-Not suitable for difficult learning material	[1, 4, 5, 14, 16, 18, 19, 23, 26, 30, 31, 35, 39, 44, 47, 50, 54, 61, 62, 63, 64, 85, 87]
Out-of-class activities (4)	-Revision of learning material -A lot of learning opportunities	-Students experience a lack of adaptive assessment	[1, 3, 4, 9, 19, 22, 31, 34, 61, 65, 71, 77, 84]
In-class activities (5)	-Use of active learning methods -A lot of learning opportunities -More interaction between students and student-teacher	-Students desire more passive explanation	[1, 3, 6, 8, 9, 19, 22, 27, 31, 34, 56, 61, 65, 66, 77, 84]

#### Self-regulated learning skills

 Several studies show factors of self-regulated learning skills that affect the effectiveness of the flipped classroom in a positive or a negative way (Table 2, section 1a). The flipped classroom prevents procrastination by completing the study material of the pre-study before the in-class activities. This results in more time for deeper learning during in-class activities. Other positive aspects relate to the student-centeredness of the learning approach. The student is responsible for his own learning and can learn at his own pace. The student can plan the pre-class activities in his own time, can compete these activities at his own pace and has the ability to look again trough the learning material. In case of a video, students can pause and review the video. The student-centeredness enables the student to develop his self-directed learning readiness. A flipped classroom makes it possible to give students timely feedback by formative testing of the self-study and providing feedback during in-class activities. This enables the student to reflect on his own learning, this is a prerequisite for self-directed learning. However, because of the student-centeredness more self-regulation is required of the learner and the flipped classroom approach may lead to non-attendance or non-preparation. This could be caused by the assumption that the completion of either the out-of-class activities only or either the in-class activities only, is sufficient to pass the course or by the high workload.

#### Prior knowledge

 Little research has been conducted on prior knowledge in relation to the flipped classroom. One study shows that prior knowledge affects the effectiveness of the flipped classroom in a negative way (Table 2, section 1b), because the flipped classroom requires more prior knowledge. The student has to acquire and understand the knowledge on his own before the in-class activities during the out-of-class activities.

#### Learning attitude

 Several studies show that aspects of students’ learning attitude affect the effectiveness of the flipped classroom in both, a negative and a positive way (Table 2, section 1c). The student’s concentration, engagement, motivation, confidence, and satisfaction are enhanced in a flipped classroom. In the flipped classroom learning takes place in an active way, during in-class activities students actively apply the knowledge acquired during the out-of-class activities by means of for example, problem-solving activities and discussions. These types of activities increase student engagement and leads to the maintenance of a high concentration during in-class activities. Chien and Hsieh [19] found that the motivational constructs intrinsic value, self-efficacy, and self-regulation significantly increase in the flipped classroom compared to the traditional classroom. This significant difference is caused by self-paced learning, self-understanding, and self-initiated learning. However, the motivational construct cognitive strategy use does not significantly increase compared to the traditional classroom. However, more motivation is required in the flipped classroom to engage in discussion and complete the out-of-class activities. Furthermore, some students don’t prefer the flipped classroom over the traditional classroom. Probably because of the effort and engagement that is required by active learning.

#### Implementation

 Several studies showed that factors of the implementation affect the effectiveness of the flipped classroom in a negative way (Table 2, section 3). Time and resources should be invested by the teacher, student, and the institute to implement the flipped classroom. Out-of-class learning content that ensures the acquiring of knowledge before class, and in-class learning activities such as cases and assignments should be created. In a lot of the flipped classrooms technology is used. Another investment that should be made to implement the flipped classroom is therefore the investment in technology.

#### Task characteristics

 Several studies showed factors of task characteristics that affect the effectiveness of the flipped classroom in a positive or a negative way (3). The flipped classroom facilitates the development of higher cognitive skills by providing guidance during in-class activities in which the higher cognitive skills are acquired. Higher cognitive skills are the three highest levels of Bloom’s taxonomy and consider the application, analysis, and synthesis of knowledge. The flipped classroom is in many cases a competence-oriented approach. Acquiring new competencies require more integrative tasks. Furthermore, problem-solving skills are enhanced in a flipped classroom. For instance, a survey conducted by Cheng et al. [18] shows that 80% of the participants experience an increase in their problem solving ability as a result of the flipped classroom. However, the flipped classroom is not suitable for learning material that is too difficult for the learner to understand by himself, because the learner must be able to achieve the lower-order learning goals by himself during the out-of-class activities.

#### Out-of-class activities

 Several studies have been conducted on out-class-activities in relation to flipped classroom (Table 2, section 40). Positive factors are that students can look again through the learning material and the flipped classroom provides a lot of learning opportunities by enabling interaction and active learning. However, a negative factor is that students experience a lack of adaptive assessment in the flipped classroom, for example a lack of feedback.

#### In-class activities

 The last factor that appeared by several studies to affect the effectiveness of the flipped classroom is in-class activities (5). A positive factor is the use of active learning methods in the flipped classroom, which provides a lot of learning opportunities in class. Another positive factor is the increase of interaction between students and between the teacher and the student. However, a negative factor is that students desire more passive explanation. Probably because students are sometimes not able to acquire the lower-order skills on their own during the out-of-class activities, when the constructs are new or difficult to understand.

#### Teacher characteristics

 The factor teacher characteristics affects the effectiveness of the flipped classroom not directly and acts only as an intermediating factor. This factor is further described in the next section.

#### Intermediating factors contributing to the effectiveness of the flipped classroom

 The second research question addressed the factors that intermediate the effect of the main factors on the effectiveness of the flipped classroom. The main factors student characteristics, task characteristics, and out-of-class activities affect the effectiveness of the flipped classroom not only directly, but also indirectly. The main factor teacher characteristics affects the effectiveness of the flipped classroom as a mediator. The main factors in-class activities and implementation affect the flipped classroom only directly and are therefore not described in this section. Table 3 shows these intermediating factors of the effectiveness of the flipped classroom per main factor.

**Table 3:** Intermediating factors of the flipped classroom

Factors	Intermediating factors of effectiveness	References
**Student characteristics**		
*Self-regulated learning skills (1a)*	-Students need to adapt their learning approach and study habits -Students need to continuously review the learning objectives -Student’s level of self-regulation	[9, 10, 43, 49, 66, 69, 74, 80, 94]
*Prior knowledge (1b)*	-Students with a lot of prior knowledge report to learn more, when seductive details are included	[53]
*Learning attitude (1c)*	-Spacing of study sessions enhances motivation -Videos enhance motivation -Students must accept the flipped classroom -Group work preference and level of engagement	[3, 4, 7, 12, 14, 20, 22, 30, 34, 35, 47, 52, 65, 72, 74, 79, 96]
Task characteristics (3)	-Little and often assessment approach -More effective for higher order tasks -Non-didactic approach -In-and out-of-class problem solving -Preliminary work before class	[48, 72, 90, 93, 94]
Out-of-class activities (4)	-Uploading materials on time -Providing guidance -Videos more effective than reading material -Multimedia modality	[3, 10, 15, 24, 47, 49, 79]
Teacher characteristics (6)	-Teachers must accept flipped classroom -Teachers role and motivation	[15, 18, 20, 22, 30, 47, 65, 66]

#### Self-regulated learning skills

 The effect of self-regulated learning skills on the effectiveness of the flipped classroom is affected by several factors (Table 3, section 1a). Students need to adapt their learning approach and study habits. The flipped classroom requires an active learning approach in which the students complete the out-of-class activities on their own and engage in active learning during in-class activities. Shibukawa and Taguchi [74] showed that students obtain significantly higher grades when they continuously review the learning objectives. In order to be effective, it is important that students have a certain level of self-regulated learning skills. Self-regulation is required for the completion of out-of-class activities. Without the completion of out-of-class activities students learn less during in-class activities.

#### Prior knowledge

 Furthermore, a factor that has been found to intermediate the effects of prior knowledge on the effectiveness of the flipped classroom, is the inclusion of seductive details (Table 3, section 1b). Maloy et al. [53] showed that students with a lot of prior knowledge report to learn more when seductive details are included. Seductive details are information that is interesting but not related to the learning objective. However, there is no difference in scores between the participants who receive seductive details and the participants who don’t receive seductive details.

#### Learning attitude

 The effect of learning attitude on the effectiveness of the flipped classroom is intermediated by several factors (Table 3, section 1c). Motivation is enhanced when videos are used and/or study sessions are spaced. Spacing is about distributing learning events in time rather than immediate succession of the learning events. Students are more satisfied when they have more shorter sessions per week than when they have less longer sessions per week. It is not yet clear what is the cause for the difference in satisfaction. In order to obtain a positive learning attitude, it is important that students accept the flipped classroom as a learning approach. This requires adaptation to the flipped classroom. Furthermore, a student’s group work preference and level of engagement affects a student’s learning attitude in the flipped classroom. This is because of the active learning methods that are used, such as group assignments.

#### Task characteristics

 Several intermediating factors are found that are related to task characteristics (Table 3, section 9). A little-and-often assessment approach enhances the engagement and achievement of the learner, because of the engagement of the learner with his learning process. The flipped classroom is more effective for higher order tasks. It is more effective for tasks such as problem-solving than for tasks such as memorizing factual knowledge. A non-didactic approach makes the flipped classroom more effective. A non-didactic approach is considered as a learning approach in which students have to reflect on the learning material in order to draw their own conclusions and consider the relevance of the different perspectives that are taken. Furthermore, in-and out-of-class problem-solving and the completion of preliminary work before class enhances the effectiveness of the flipped classroom.

#### Out-of-class activities

 The effect of out-of-class activities on the effectiveness of the flipped classroom is also mediated by several intermediating factors (Table 3, section 40). It is important to upload the materials on time to make sure that the students have sufficient time to complete the out-of-class assignments. During the out-of-class activities, guidance should be provided. It is not yet known how this guidance ideally looks like. Videos are more effective than reading materials.

#### Teacher characteristics

 Other intermediating factors that have been found relate to teacher characteristics (Table 3, section 6). Teachers must accept the concept of flipped classroom. The role of the teacher changes from lecturer to coach, facilitator, and mentor who supports the student’s learning process. Furthermore, the motivation of the teacher for teaching is important.

#### Interventions stimulating the affecting factors

 The third research question addressed the interventions that can be used to stimulate the effecting factors in a positive way. The main factors affecting the effectiveness of the flipped classroom directly and/or indirectly as a mediating factor could be stimulated by several interventions. Table 4 shows these interventions, categorized by the main factors.

**Table 4:** Interventions to stimulate the affecting factors positively

Factors	Intervention	References
**Student characteristics**		
*Self-regulated learning skills* (1a)	-Encourage class attendance and participation -Provide possibilities to support the development of self-regulated learning skills, such as prompts or feedback -Provide the students structure by organizing the learning process into stages and by giving a guideline -Use a detailed rubric to show students their progress and how they can develop -Give a small proportion of the course grade to formative assessment to show that you value it -Manage the working load of students by making homework optional -Be adaptive and provide just-in-time interventions	[8, 10, 13, 34, 36, 43, 47, 48, 52, 64, 68, 69, 74, 79, 84, 96, 98]
*Learning attitude* (1c)	-Create a performance-approach environment -Make videos more engaging by creating an interactive authentic environment -Consider the preview of learning materials, interaction with peers, teacher facilitation, and classroom participation -Make the curriculum relevant for students -Make use of simulation	[20, 34, 48, 51, 80, 86]
Implementation (2)	-Make use of existing materials -Pay as much attention to grades as to student perception -Realize that different learning and teaching approaches are needed in the flipped classroom -Address expectations -Integrate active learning methodologies into a large number of subjects -Use the flipped classroom at least 3 weeks -Implement the flipped classroom gradually -Provide sufficient support	[8, 10, 14, 34, 52, 56, 57, 64, 75, 80, 93, 96]
Task characteristics (3)	-Include in-and out-of-class problem solving activities	[90]
Out-of-class activities (4)	-Instruct students to work together with peers -Structure the goals, learning methods, and assessment -Make use of e-learning, quizzes, learning management systems, gamification, digital workbooks, online micro lectures, and mobile-based learning -Let students produce their own materials -Online materials should always be available -Take care adding text information on videos -Include authentic assessment to assess the acquired knowledge	[2, 11, 36, 38, 39, 41, 45, 46, 68, 83, 87, 97]
In-class activities (5)	-Make use of group management -Structure the goals, teaching methods, and assessment -Make use of a partially flipped classroom -Consider the importance of student satisfaction for the scheduling -Review key concepts at the beginning of class -Create opportunities for dialogue	[12, 35, 42, 56, 64, 84, 96]
Teacher characteristics (6)	-Pay attention to relevant dimensions of flipped classroom in teacher training	[40]

#### Self-regulated learning skills

 Several interventions can be done to stimulate the self-regulated learning skills (Table 4, section 1a). First of all, it is important to encourage class attendance and participation and to provide students possibilities to support the development of self-regulated learning skills. The development of self-regulated learning skills could be supported by providing prompts or feedback. Examples of prompts are to ask the students to relate the new knowledge to prior knowledge or to make the concepts visual. Feedback can help students to adjust their learning. A detailed rubric and formative assessment can also help to show students their progress and how they can develop. To show that formative assessment is valued, a small proportion of the course grade can be given to the formative assessment. The student can be provided structure by organizing the learning process into stages and providing students a guideline. A student’s working load can be higher in a flipped classroom compared to a traditional classroom. In order to manage the working load, home-work can be made optional. Furthermore, it is important to be adaptive as a teacher and to provide just-in-time interventions to make learning optimal and meet the needs of the individual learner.

#### Learning attitude

 The learning attitude can also be positively stimulated by several interventions (Table 4, section 1c). It is important to create a performance-approach environment. Learners who have a performance-approach strive to be the best or be seen as talented. The performance-approach is significantly related to achievement and self-regulated learning. By creating a high competitiveness context, the performance-approach is stimulated. Videos can be made more engaging by creating an interactive authentic environment. This can be done by making a video in which the teacher interacts with the student. This enhances a student’s engagement. Consider the preview of learning materials, interaction with peers, teacher facilitation, and classroom participation in the design of the flipped classroom. Preview of learning materials, teaching facilitation, and classroom participation positively predict enjoyment. Cho et al. (2021) conclude: “students experienced enjoyment if they (a) perceived the preview materials as meaningful and helpful, (b) believed the instructors facilitated their learning, and (c) actively participated in learning activities” (p. 9). Furthermore, it is important to make the curriculum as relevant as possible for students.

#### Implementation

 Several interventions can be done to stimulate the implementation (Table 4, section 3). To spare time and resources, existing materials can be used. Blair et al. [8] recommend paying as much attention to student grades as to student perception during the implementation of the flipped classroom. They state that it is important to realize that the flipped classroom requires different teaching and learning approaches in order to prevent decrease of grades because of the requirement of self-regulation and change of learning approach. The teachers’ and students’ expectations should be addressed at the beginning of the implementation. To get used to the flipped classroom it is important to integrate active learning methodologies into a large number of subjects. Use the flipped classroom at least three weeks and implement it gradually to facilitate the adjustment of the learner to the flipped classroom.

#### Task characteristics

 In regard to task characteristics, little research has been conducted on interventions to address this (Table 4, section 9). Although, F. H. Wang [90] mentioned that it is important to include in-and out-of-class problem solving tasks in a flipped classroom to develop the problem solving ability.

#### Out-of-class activities

 Several interventions have been found that stimulate out-of-class activities (Table 4, section 40). Instruct students to work together with peers during the out-of-class activities and provide the student with a good structure by structuring the goals, learning method, and assessment. Make use of out-of-class activities that fit the students well by making use of learning methods such as e-learning, quizzes, learning management systems, gamification, and mobile-based learning. Students can also produce their own materials by making use of online tools. The online materials should always be available. Take care adding text information on the videos because of the cognitive load. The knowledge acquired during the out-of-class activities can be assessed by making use of authentic assessment.

##### In-class activities

 The studies also show several interventions related to in-class activities (Table 4, section 5). Group management can be used to make the in-class activities more effective. With group management the group is divided into small groups, each group containing one student with a high Grade Point Average, who can help their group members when needed. Just like the out-of-class activities it is important to provide structure during in-class activities by structuring the goals, teaching method, and assessment. A partially flipped classroom can be used to make sure that the students adapt more easily to the flipped classroom. To increase the student satisfaction, it is important to space the sessions and to make sure that all students understand the constructs discussed in the out-of-class activities the key constructs could be reviewed at the beginning of the in-class activities. Furthermore, it is important to create opportunities for dialogue.

##### Teacher characteristics

Furthermore, in regard to teacher characteristics, little research has been conducted on interventions to address this (Table 4, section 6). Hyypiä et al. [40] mention that it is important to pay attention to relevant dimensions of the flipped classroom in teacher training. Relevant dimensions of the concept of flipped classroom are pedagogical practices, curriculum work, students’ guidance and counseling, technology, and assessment [40].

## Discussion

### Aim

 This state-of-the-art-review aimed to investigate the current state of knowledge about factors contributing to the effectiveness of the flipped classroom and interventions to positively affect these factors. This review has qualitatively analyzed the studies that are conducted on the instructional method flipped classroom in the health science education domain from 2016 till 2020. This study aimed to answer the following question: Which factors affect the effectiveness of the flipped classroom and how could these factors be positively stimulated? In order to answer this question the following sub questions will be answered: 1) What are positive and negative factors of the flipped classroom? 2) Which factors mediate the effectiveness of the flipped classroom? 3) How could the factors be stimulated in a positive way?

### Primary findings

 This study showed that six main factors play a role in the effectiveness of the flipped classroom: student characteristics, teacher characteristics, implementation, task characteristics, out-of-class activities and in-class activities. The factor student characteristics consists of the sub-factors self-regulated learning skills, prior knowledge, and learning attitude. The factors student characteristics, implementation, task characteristics, out-of-class activities, and in-class activities affect the effectiveness of the flipped classroom in a positive and/or a negative way, shown in Table 2. The effect of these factors on the effectiveness of the flipped classroom is mediated by the factors student characteristics, teacher characteristics, task characteristics, and out-of-class activities, shown in Table 3. The affecting and intermediating factors can be stimulated by several interventions, shown in Table 4.

### Findings related to earlier research

 Our study showed that learning attitude can affect the effectiveness of the flipped classroom negatively, because not all students prefer the flipped classroom. This is not consistent with a previous study conducted by Hew and Lo [37]. They showed that students prefer flipped classroom over traditional classroom. This could be caused by the design of the flipped classroom. Our study showed that learning attitude can be positively enhanced by making the curriculum relevant for the students.

In the literature there is no consistence about whether the flipped classroom enhances students’ study achievement. The meta-analysis conducted by Chen et al. [17] showed that student achievement is higher in a flipped classroom compared to a traditional classroom. However, the meta-analysis conducted by Gillette et al. [29] showed no significant difference in test scores between the flipped classroom and the traditional classroom. This could be due to a difference in design of the flipped classroom between the included studies of both meta-analyses. The studies did not use a standardized flipped classroom format and the design differed a lot between the studies. To enhance the effectiveness of the flipped classroom it is important to consider the factors that directly and indirectly affect the effectiveness of the flipped classroom during the design of the flipped classroom, as described in this study.

Our study showed that an intermediating factor of the effect of prior knowledge on the effectiveness of the flipped classroom is the inclusion of seductive details. Students with more prior knowledge learn more when seductive details are included, whereas students with fewer prior knowledge learn less when seductive details are included. This is in line with the cognitive load theory, as developed by Sweller [81]. The positive factors of the flipped classroom regarding self-regulated learning, the student can learn at his own pace, is responsible for his own learning, and develops self-directed learning readiness, is in line with the learning theory of constructivism. The intervention of creating an authentic environment is also in line with constructivism.

A meta-analysis conducted by van Alten et al. [88] showed several factors that are important for a flipped classroom in order to be effective compared to a traditional classroom. The flipped classroom should contain quizzes and the face-to-face class time should be the same as in the traditional classroom [88]. Our study also showed quizzes as one of the many interventions to enhance the effectiveness of the flipped classroom. To stimulate the factors affecting the flipped classroom in a positive and/or a negative way, the interventions mentioned in this study could be applied.

### Strengths of the review

 The research team consists of policy advisors and educational researchers. Therefore, different perspectives on education come together in this research. This makes the review multi perspective. The review had a broad scope, because all factors of the flipped classroom are taken into account in this review. This provides a great overview of the factors that are involved in the effectiveness of the flipped classroom.

### Limitations of the review

 This review also has limitations that should be addressed. For instance, factors affecting the effectiveness of the flipped classroom in a positive or a negative way were derived from the qualitative analysis. However, whether an effect is positive, negative is relative. This study considers the fact that not all students prefer the flipped classroom over a traditional classroom as a negative effect. Discussing this with colleagues, we came to the conclusion that this could also be seen as a given, that is neutral.

Another limitation of this study is that some findings could have more value than other findings because of the design and the quality of the included study. As described in the method section, 11 of the 88 included studies were assessed as insufficient. A lot of the studies compare the effectiveness of the flipped classroom to the traditional classroom. However, some studies did not include a control group. Some studies were randomized controlled trials, whereas other studies were quasi-experimental or qualitative studies. Furthermore, to some aspects of the flipped classroom (for example the aspect prior knowledge and task characteristics) less research is conducted than to other aspects of the flipped classroom (for example self-regulated learning or in-class activities). This could have implications for the value of the findings.

### Recommendations for further research

 To several aspects of the flipped classroom a lot of research has been conducted, for example on in-class activities and self-regulated learning. However, on the aspects prior knowledge, teacher characteristics, and task characteristics little research has been conducted. Therefore, future research should be conducted on these aspects. An effect study could be conducted to the different interventions described in this study. It is not known yet how much effect these interventions have on the effectiveness of the flipped classroom. Therefore, a pre-post control group intervention study should be conducted to evaluate the effectiveness of these interventions. Another suggestion for future research could be the investigation of how the guidance during out-of-class activities ideally looks like. Research has shown that guidance during out-of-class activities should be provided in order to make the flipped classroom effective. For example, in the form of prompts and feedback, as described at page 16. However, it is not yet known how this guidance should be designed in order to be most effective. Furthermore, future research should be conducted to investigate whether the findings of this review can be generalized to other domains than health science education.

## Conclusion and practical implications

 We can conclude that by applying the interventions for an effective flipped classroom and taking the factors affecting the effectiveness into account in the design of the flipped classroom, student learning can be stimulated in the flipped classroom. This study provides interventions for teachers, educational designers, and boards of universities to effectively use the flipped classroom. By applying the flipped classroom and enhancing the effectiveness of the flipped classroom by applying these interventions, the student- centeredness, efficiency, and flexibility of health science education will be enhanced. This way students can more effectively and efficiently master the competencies that are needed to become a good health professional.

## Supplementary Information

**Additional file 1. **Study characteristics and quality.

## Data Availability

Authors can confirm that all relevant data are included in the article and/or its additional files.

## Abbreviation

PRISMA: Preferred Reporting Items for Systematic Reviews and Meta-analyses
